# Association between malnutrition and prognosis in colorectal cancer: a systematic review and meta-analysis

**DOI:** 10.3389/fonc.2026.1789366

**Published:** 2026-05-08

**Authors:** Chao Gao, Xiu-Ning Zhang

**Affiliations:** 1 Department of Gastroenterology, Xiongan Xuanwu Hospital, Xiongan New Area, Baoding, China; 2 Department of Digestive Endoscopy, Hebei Provincial Hospital of Traditional Chinese Medicine, Shijiazhuang, Hebei, China

**Keywords:** colorectal cancer, GLIM, global leadership initiative on malnutrition, malnutrition, overall survival

## Abstract

**Background:**

Malnutrition is prevalent in colorectal cancer (CRC) and may adversely influence oncologic prognosis and perioperative recovery. This systematic review and meta-analysis quantified associations between malnutrition diagnosed by the Global Leadership Initiative on Malnutrition (GLIM) criteria and clinical outcomes in CRC.

**Methods:**

This study followed the Preferred Reporting Items for Systematic Reviews and Meta-Analyses (PRISMA) statement. PubMed, Embase, Web of Science Core Collection, and The Cochrane Library were searched through December 31, 2025. Observational cohort studies enrolling adults with CRC and reporting outcomes according to GLIM-defined malnutrition were included. Study quality was assessed using the Newcastle–Ottawa Scale. Pooled hazard ratios (HRs), risk ratios (RRs), and mean differences (MDs) with 95% confidence intervals (CIs) were synthesized using random-effects models.

**Results:**

Nine cohort studies involving 4,771 patients were included. GLIM-defined malnutrition was associated with poorer overall survival (HR 1.23, 95% CI 1.12–1.33; I² = 56.4%). It was also associated with increased postoperative infectious complications (RR 1.61, 95% CI 1.51–1.98; I² = 0.0%), major complications (RR 1.36, 95% CI 1.18–1.60), overall postoperative complications (RR 1.44, 95% CI 1.25–1.56), 30-day mortality (RR 2.03, 95% CI 1.35–3.31), and 30-day readmission (RR 1.34, 95% CI 1.01–1.64). Malnutrition was further associated with prolonged length of hospital stay (MD 2.30 days, 95% CI 1.40–3.10). Sensitivity and subgroup analyses supported the robustness of the findings.

**Conclusions:**

GLIM-defined malnutrition is associated with inferior survival, increased postoperative morbidity, and greater healthcare utilization in CRC. These findings support routine nutritional screening followed by GLIM-based assessment for perioperative risk stratification and clinical management in this population.

**Systematic review registration:**

https://www.crd.york.ac.uk/prospero/, identifier CRD420261324729.

## 1 Introduction

Colorectal cancer (CRC) remains a leading cause of cancer morbidity and mortality and is commonly managed using multimodal strategies, including curative-intent surgery, perioperative systemic therapy, and radiotherapy in selected settings. Despite advances in oncologic treatment, outcomes vary substantially and are influenced by host factors that affect treatment tolerance and recovery (1, 2). Malnutrition is clinically relevant in CRC because it is common and reflects a catabolic state characterized by immune dysfunction, dysregulated systemic inflammation, diminished physiologic reserve, and skeletal muscle wasting (sarcopenia) (3). In large international surgical datasets, malnutrition has been associated with worse early postoperative outcomes after cancer surgery, supporting its importance as a modifiable perioperative risk factor (4, 5). A major challenge in nutritional oncology is the heterogeneity in how nutritional risk and malnutrition are operationalized and measured, particularly the distinction between risk screening and formal diagnostic classification, which hampers cross-study comparability (6). Traditional instruments frequently used in oncology and surgical practice, such as the Nutritional Risk Screening 2002 (NRS-2002) (7), Patient-Generated Subjective Global Assessment (PG-SGA) (8), and Malnutrition Universal Screening Tool (MUST) (9), primarily identify nutritional risk rather than establish a standardized diagnostic classification of malnutrition. This conceptual distinction has been highlighted in prior methodological studies and consensus statements, which note that reliance on heterogeneous screening tools may lead to inconsistent prevalence estimates and variable prognostic associations across studies. The Global Leadership Initiative on Malnutrition (GLIM) criteria were developed to provide a consensus-based diagnostic framework that integrates phenotypic and etiologic domains, thereby standardizing malnutrition diagnosis across clinical settings (10). Recent evidence across cancer populations indicates that GLIM-defined malnutrition is associated with inferior survival and increased postoperative complications, although the certainty of evidence and the magnitude of association vary by cancer type and clinical context (11, 12).

In CRC, several contemporary cohort studies have examined GLIM-defined malnutrition in relation to both short-term surgical outcomes and long-term prognosis (13). Data in elderly surgical cohorts suggest that GLIM-defined malnutrition correlates with postoperative complications and overall survival, emphasizing clinical relevance in populations with limited physiologic reserve (14). Additional studies have incorporated functional and body-composition measures, such as handgrip strength and computed tomography–derived skeletal muscle indices, to refine nutritional phenotyping and risk stratification, reflecting the intersection between malnutrition, sarcopenia, and cancer outcomes in CRC (15, 16). However, reported prevalence estimates, screening approaches preceding GLIM classification, treatment settings, and follow-up durations differ across cohorts, which may contribute to between-study variability and limit direct clinical translation of individual findings (17). From a clinical practice perspective, contemporary perioperative nutrition guidance emphasizes structured assessment and targeted nutritional optimization as part of surgical care pathways, reinforcing the need for evidence that links standardized diagnostic frameworks to clinically meaningful endpoints (18). Against this background, a focused synthesis of CRC-specific evidence is warranted to quantify the prognostic value of GLIM-defined malnutrition and to contextualize heterogeneity across study designs and clinical settings (19). Notably, while broad oncology meta-analyses support an adverse prognostic signal for GLIM-defined malnutrition, CRC-specific estimates are needed because baseline risk, treatment intensity, complication profiles, and competing causes of mortality vary by tumor site and care pathway (20, 21).

Therefore, this systematic review and meta-analysis aimed to evaluate the association between GLIM-defined malnutrition and prognosis in CRC, with emphasis on time-to-event outcomes and postoperative morbidity. By synthesizing cohort evidence and exploring prespecified sources of heterogeneity, this study sought to clarify the clinical implications of applying a standardized malnutrition diagnosis in CRC and to inform risk stratification and supportive care planning across perioperative and longitudinal management.

## 2 Methods

### 2.1 Search strategy

We conducted this systematic review and meta-analysis in accordance with the Preferred Reporting Items for Systematic Reviews and Meta-Analyses (PRISMA) statement (22). The protocol was prospectively registered in PROSPERO (Registration No. CRD420261324729). The literature search and study selection were independently conducted by CG and XNZ. Reference management software (EndNote) was used to assist in duplicate removal and record management; all eligibility decisions were verified manually. A comprehensive literature search was performed in PubMed, Embase, Web of Science Core Collection, and The Cochrane Library to identify cohort studies evaluating the association between malnutrition defined by the GLIM criteria and prognosis in colorectal cancer. The databases were searched through December 31, 2025, with the final search conducted on that date; studies published up to this date were eligible for inclusion. No restrictions on language or publication status were applied. Controlled vocabulary (MeSH in PubMed; Emtree in Embase) was combined with free-text terms related to colorectal cancer, GLIM-based malnutrition, and prognostic outcomes. Reference lists of included articles and relevant reviews were manually screened to identify additional eligible studies, and supplementary searches of related resources were conducted when necessary. The detailed search strategies for each database are presented in **Supplementary Table 1**.

### 2.2 Inclusion criteria and exclusion criteria

#### 2.2.1 Inclusion criteria

1. Population: Adults aged 18 years or older with colorectal cancer, including colon and rectal cancer, confirmed by histopathology or clearly defined clinical criteria; mixed cancer cohorts were eligible only when colorectal cancer–specific data were extractable.
2. Exposure: Malnutrition diagnosed using the GLIM criteria, with explicit reporting of GLIM-based classification and sufficient methodological description to support categorization.
3. Comparator: Patients without GLIM-defined malnutrition or comparisons across GLIM severity categories with extractable contrasts.
4. Outcomes: At least one prognostic outcome, including overall survival, disease-free survival, recurrence-free survival, progression-free survival, cancer-specific survival, all-cause mortality, recurrence, or metastasis; studies had to report effect estimates with confidence intervals or provide sufficient data for calculation.
5. Study design: Prospective or retrospective observational cohort studies with longitudinal follow-up and clearly defined outcome ascertainment.

#### 2.2.2 Exclusion criteria

1. Exposure not eligible: Malnutrition not defined using GLIM criteria or GLIM-based classification not verifiable from the report.
2. Population not eligible: Participants younger than 18 years; mixed tumor populations without separable colorectal cancer–specific analyses; non-human studies, including animal, *in vitro*, or mechanistic research.
3. Design or publication type not eligible: Randomized trials, cross-sectional studies, conventional case–control studies, case reports, case series, conference abstracts without full text, editorials, commentaries, letters without original extractable data, narrative reviews, systematic reviews, meta-analyses, and protocols without outcomes.
4. Insufficient data: No prespecified prognostic outcomes; effect estimates not reported and not derivable from available data.
5. Overlapping cohorts: Duplicate datasets or overlapping populations, retaining only the most informative report based on sample size, follow-up duration, and completeness of adjusted analyses.

### 2.3 Data extraction

Data extraction was conducted using a prespecified standardized form. CG and XNZ independently extracted data from each eligible study, and discrepancies were resolved through discussion and consensus. Extracted data were cross-checked for accuracy. The following information was collected: first author, publication year, study design, country or region, sample size, treatment modality, follow-up duration, baseline age, sex distribution, and tumor location. We also extracted information on nutritional assessment, including the screening tool and corresponding cut-off value, as well as the prevalence of malnutrition and related nutritional variables reported by the original studies.

### 2.4 Quality assessment

Study quality was assessed using the Newcastle–Ottawa Scale (NOS) for cohort studies. CG and XNZ independently evaluated each included study, and disagreements were resolved through discussion until consensus was reached. The NOS assesses methodological quality across three domains: selection of cohorts, comparability of groups, and ascertainment of outcomes, with a maximum score of nine points. Studies were rated according to the number of stars awarded in each domain, and overall quality was categorized *a priori* as low (0–3), moderate (4–6), or high (7–9) quality. The comparability domain was considered met when analyses adjusted for major prognostic factors relevant to colorectal cancer outcomes (23).

### 2.5 Statistical analyses

All meta-analyses were conducted using Stata version 18.0 (StataCorp, College Station, TX, USA). Time-to-event outcomes were synthesized as hazard ratios with 95% confidence intervals, and dichotomous outcomes were pooled as risk ratios with 95% confidence intervals. Reported effect estimates were converted to the natural logarithmic scale, and standard errors were calculated from the corresponding confidence intervals. When both crude and adjusted estimates were available, the most fully adjusted model was used for the primary analyses. Between-study heterogeneity was assessed using Cochran’s Q test and quantified with the I² statistic. Given that all included studies were observational cohort studies conducted in different clinical settings and populations, random-effects models were used for all pooled analyses to account for both within-study and between-study variability. Pooled estimates were generated using inverse-variance weighting, and random-effects pooling was performed with the DerSimonian–Laird method.

Subgroup analyses were prespecified according to study design, screening approach (NRS-2002 at least 3 versus other or not reported), tumor location (rectum-only versus mixed colorectal cohorts), and study quality (Newcastle–Ottawa Scale 9 versus 8), with subgroup differences assessed statistically. Sensitivity analyses were performed using a leave-one-out approach. Publication bias was evaluated by funnel plot inspection and Egger’s regression test. All tests were two-sided, with p < 0.05 considered statistically significant.

## 3 Results

### 3.1 Search results and study selection

**Figure 1:**
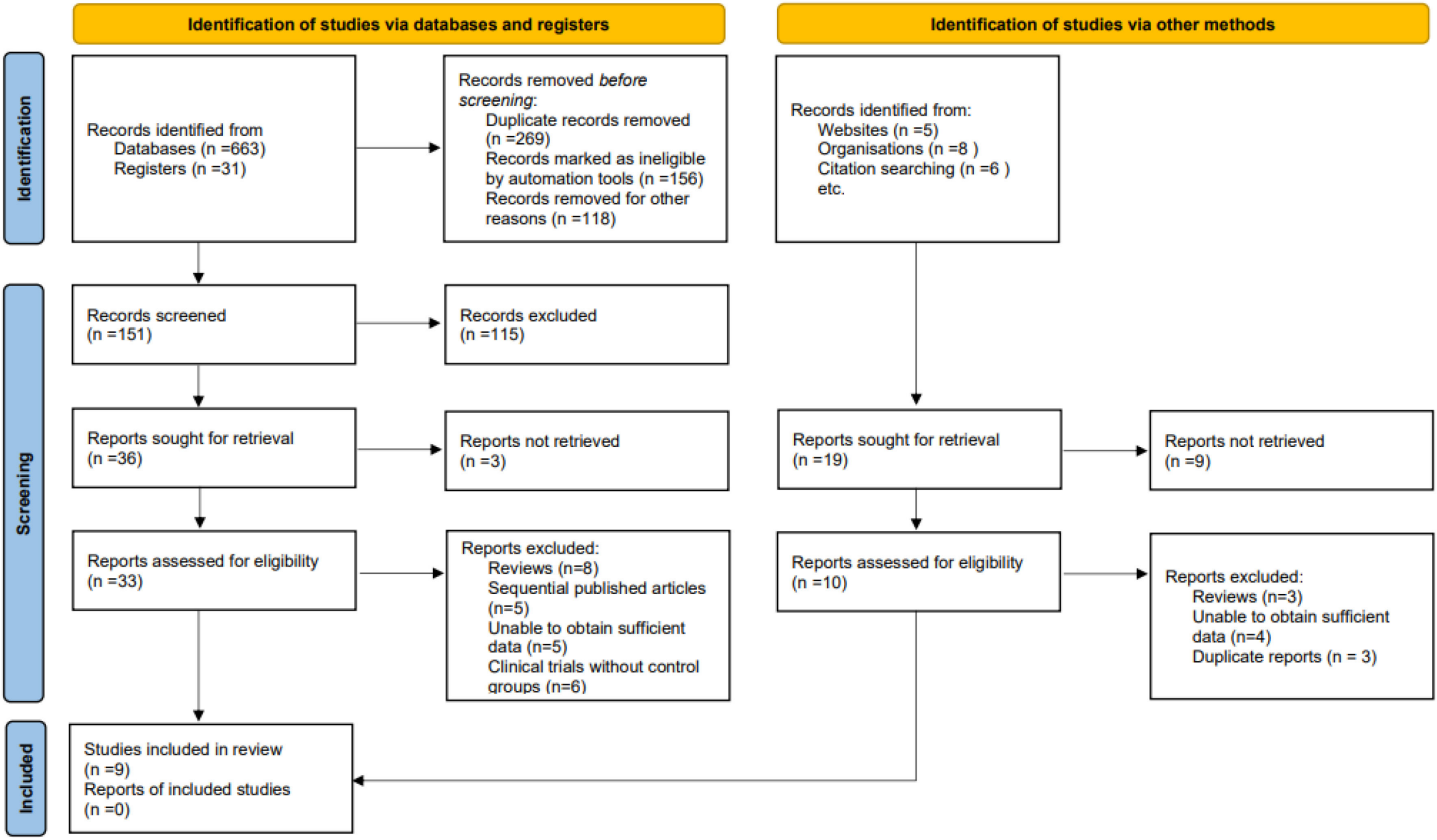
PRISMA flow diagram depicting the literature search, screening, eligibility assessment, and final inclusion of studies in the systematic review and meta-analysis.

The study selection process is shown in **Figure 1** and followed the PRISMA 2020 framework. A total of 694 records were identified from databases (n = 663) and registers (n = 31). After removal of 269 duplicates and 118 records for other reasons, 151 records underwent title and abstract screening, of which 115 were excluded. Thirty-six reports were sought for full-text review; three could not be retrieved, leaving 33 articles assessed for eligibility. Twenty-four reports were excluded due to being reviews (n = 8), sequential publications (n = 5), insufficient data (n = 5), or clinical trials without control groups (n = 6). An additional 19 records were identified through other sources (websites, n = 5; organizations, n = 8; citation searching, n = 6). Of these, nine could not be retrieved and ten underwent full-text assessment; all were excluded due to being reviews (n = 3), insufficient data (n = 4), or duplicate publications (n = 3). Ultimately, nine studies met the eligibility criteria and were included in the qualitative and quantitative synthesis (24–31).

### 3.2 Study characteristics

**Table 1** summarizes nine cohort studies comprising a total of 4,771 patients with colorectal cancer. Six studies were retrospective in design, and three were prospective cohort studies. Sample sizes ranged from 121 to 1,358 participants. Across studies, the mean or median age of participants generally ranged from 60 to 66 years, indicating that most cohorts included patients in the sixth to seventh decade of life. The proportion of male participants ranged from approximately 53% to 63% in most studies. Tumor sites included colon and rectal cancers, with three studies restricted to rectal cancer populations and the remainder enrolling mixed colorectal cohorts. Treatment strategies were predominantly surgery-based, with several studies incorporating multimodal therapies such as chemotherapy, radiotherapy, immunotherapy, or perioperative nutritional support. Follow-up duration varied according to outcome type. Postoperative complication endpoints were typically assessed within 30 to 90 days, whereas survival outcomes were evaluated over longer follow-up periods ranging from 5 to 8 years. Nutritional risk screening was most commonly performed using NRS-2002 (reported in six studies), while one study used PG-SGA Short Form (PG-SGA SF), and screening information was not reported in the remaining studies. The prevalence of GLIM-defined malnutrition varied substantially across studies, ranging from 12.4% to 54.8%, reflecting differences in patient populations and operational definitions.

**Table 1:** Characteristics of included cohort studies and nutritional assessment methods in colorectal cancer.

Author	Year	Study design	Sample size (n)	Age (as reported)	Male, n (%)	Screening positive, n (%)	GLIM-defined malnutrition, n (%)	Alternative GLIM operationalization/other GLIM categories	BMI data (kg/m²), as reported	Treatment modality	Follow-up and endpoints	Tumor location	Screening tool and cut-off
Dos Santos et al. (27)	2024	Retrospective cohort study	208	61.0 (53.0, 69.0)	139 (53.3)	NR	114 (54.8)	—	25 (12.0)	Radical colorectal surgery (laparotomy/laparoscopy), adjuvant/neoadjuvant therapy	1 year: OS; during hospitalization: MC	Rectum 51.3%; colon 48.7%	Not reported
Yildiz Kopuz et al. (30)	2024	Prospective cohort study	121	62.3 ± 12.08	76 (62.8)	NR	55 (45.5)	—	27.1 ± 5.09	Surgical treatment	30 days: MC	Colon 41.3%; rectum 58.7%	NRS-2002 ≥3
Da Silva Couto et al. (26)	2024	Retrospective cohort study	191	Well-nourished 59.70 ± 11.29; malnourished 63.20 ± 11.15	110 (57.5)	NR	63 (33.0)	—	111 (12.1)	Chemotherapy and/or radiotherapy	5 years: OS	Colon 51.3%; rectosigmoid 7.9%; rectum 40.8%	PG-SGA SF ≥3
Chen et al. (24)	2024	Retrospective analysis within a prospective registered study (ChiCTR2200057818)	850	61.0 (53.0, 69.0)	139 (53.3)	184 (21.6)	105 (12.4)	HGS-GLIM: 54 (6.4)	Median 25.12	Radical surgery; preoperative nutritional supplementation for NRS-2002 ≥3	30 days: MC; 8 years: OS	Rectum 51.3%; colon 48.7%	NRS-2002 ≥3
Zhou et al. (31)	2023	Retrospective study	318	65 (58–74)	379 (60.7)	NR	114 (18.3)	WN: 204 (32.7); WO: 264 (42.3)	<18.5: 72 (11.5); 18.5–23.9: 362 (58.0)	LAR (Dixon) or APR (Miles)	30 days: IC; 6 years: OS	Rectum 100%	NRS-2002 ≥3
Zhou et al. (31)	2022	Prospective study	171	64.59 ± 9.55	96 (56.4)	NR	63 (36.0)	—	44 (25.73)	Radical colorectal surgery (laparotomy/laparoscopy)	90 days: IC	Rectum 50.2%; colon 49.7%	NRS-2002 ≥3
Song et al. (29)	2022	Prospective cohort study	918	66 (17)	555 (60.5)	310 (33.8)	217 (23.6)	—	111 (12.1)	Radical surgery for CRC (laparotomy/laparoscopy)	30 days: MC, IC; 6 years: OS	Not reported	NRS-2002 ≥3
Chen et al. (25)	2022	Retrospective study	636	65 (IQR 17)	385 (60.5)	186 (29.2)	158 (24.8)	MO: 42 (6.7)	≥24: 190 (30.5); 99 (15.6)	Proctectomy; resection/APR	During hospitalization: MC; 8 years: OS	Rectum 100%	NRS-2002 ≥3
Ruan et al. (28)	2022	Retrospective analysis of a multicenter prospective observational cohort	1358	60 (52–67)	810 (59.7)	NR	345 (25.41)	—	175 (19.51)	Surgery, chemotherapy, radiotherapy, immunotherapy, nutritional support	6 years: OS	Colon 47.35%; rectum 52.65%	Not reported

APR, abdominoperineal resection; BMI, body mass index; CRC, colorectal cancer; GLIM, Global Leadership Initiative on Malnutrition; HGS, handgrip strength; IC, infectious complications; IQR, interquartile range; LAR, low anterior resection; MC, major complications; MN, malnourished (as reported in the original study); MO, malnourished obesity (as reported in the original study); NRS-2002, Nutritional Risk Screening 2002; NR, not reported; OS, overall survival; PG-SGA SF, Patient-Generated Subjective Global Assessment Short Form; WN, well-nourished (as reported in the original study); WO, well-nourished overweight or at-risk category as reported by the original study.

### 3.3 Methodological quality of included studies

Methodological quality was evaluated using the Newcastle–Ottawa Scale for cohort studies (**Table 2**). Overall, the included studies demonstrated high methodological quality, with total NOS scores ranging from 8 to 9 points out of a maximum of 9. Most studies achieved full scores in the selection domain, indicating generally appropriate cohort representativeness, clear selection of comparison groups, and robust ascertainment of exposure, together with confirmation that the outcomes of interest were not present at baseline. The comparability domain was also largely well addressed, as the majority of studies received the maximum comparability score, suggesting that key confounders were considered in the design or statistical analyses. For the outcome domain, outcome assessment and adequacy of follow-up were generally satisfactory. A small proportion of studies did not receive a star for the “follow-up long enough” item, reflecting variability in follow-up duration relative to the outcomes evaluated. Collectively, these findings indicate a low risk of major bias across the included cohorts and support the reliability of the pooled estimates.

**Table 2:** The quality assessment according to Newcastle-Ottawa scale of each cohort study.

Study	Representativeness of the exposed cohort	Selection of the non-exposed cohort	Ascertainment of exposure	Demonstration that outcome Of interest was not present at start of study	Comparability of cohorts on the basis of the design or analysis	Assessment of outcome	Was follow-up long enough	Adequacy of follow up of cohorts	Total score
Dos Santos et al.	1	1	1	1	1	1	1	1	8
Yildiz Kopuz et al.	1	1	1	1	2	1	1	1	9
Da Silva Couto et al.		1	1	1	2	1	1	1	8
Chen et al.	1	1	1	1	2	1		1	8
Zhou et al.	1	1	1	1	2	1	1	1	9
Zhou et al.	1	1	1	1	2	1	1	1	9
Song et al.	1	1	1	1	2	1		1	8
Chen et al.	1	1	1	1	2	1	1	1	9
Ruan et al.	1	1	1	1	1	1	1	1	8

### 3.4 Overall survival and sensitivity analysis

**Figure 2:**
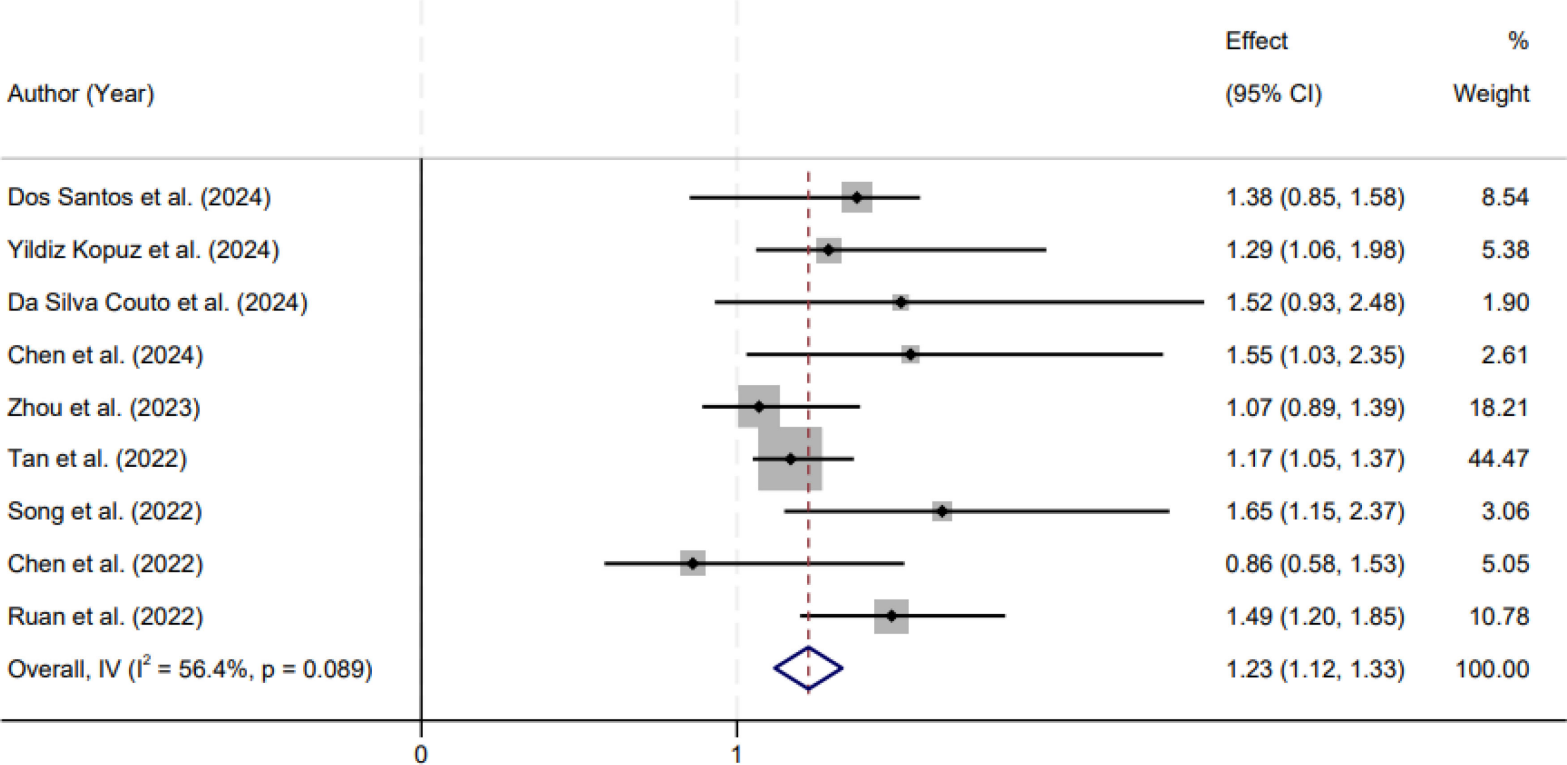
Forest plot showing the pooled association between GLIM-defined malnutrition and overall survival in patients with colorectal cancer using a random-effects model.

Nine cohort studies were included in the quantitative synthesis of overall survival. Between-study heterogeneity was moderate and approached statistical significance (I² = 56.4%, Cochran’s Q test p = 0.089); therefore, a random-effects model was applied to generate a conservative pooled estimate that accounted for inter-study variability. The meta-analysis showed that GLIM-defined malnutrition was associated with poorer overall survival. Specifically, patients without malnutrition according to the GLIM diagnostic framework experienced significantly longer overall survival than those classified as malnourished, with a pooled hazard ratio of 1.23 (95% confidence interval 1.12–1.33) (**Figure 2**). This finding indicates that GLIM-defined malnutrition confers an approximately 23% higher hazard of death during follow-up relative to the non-malnourished group, supporting the prognostic relevance of standardized malnutrition diagnosis in colorectal cancer.

**Figure 3:**
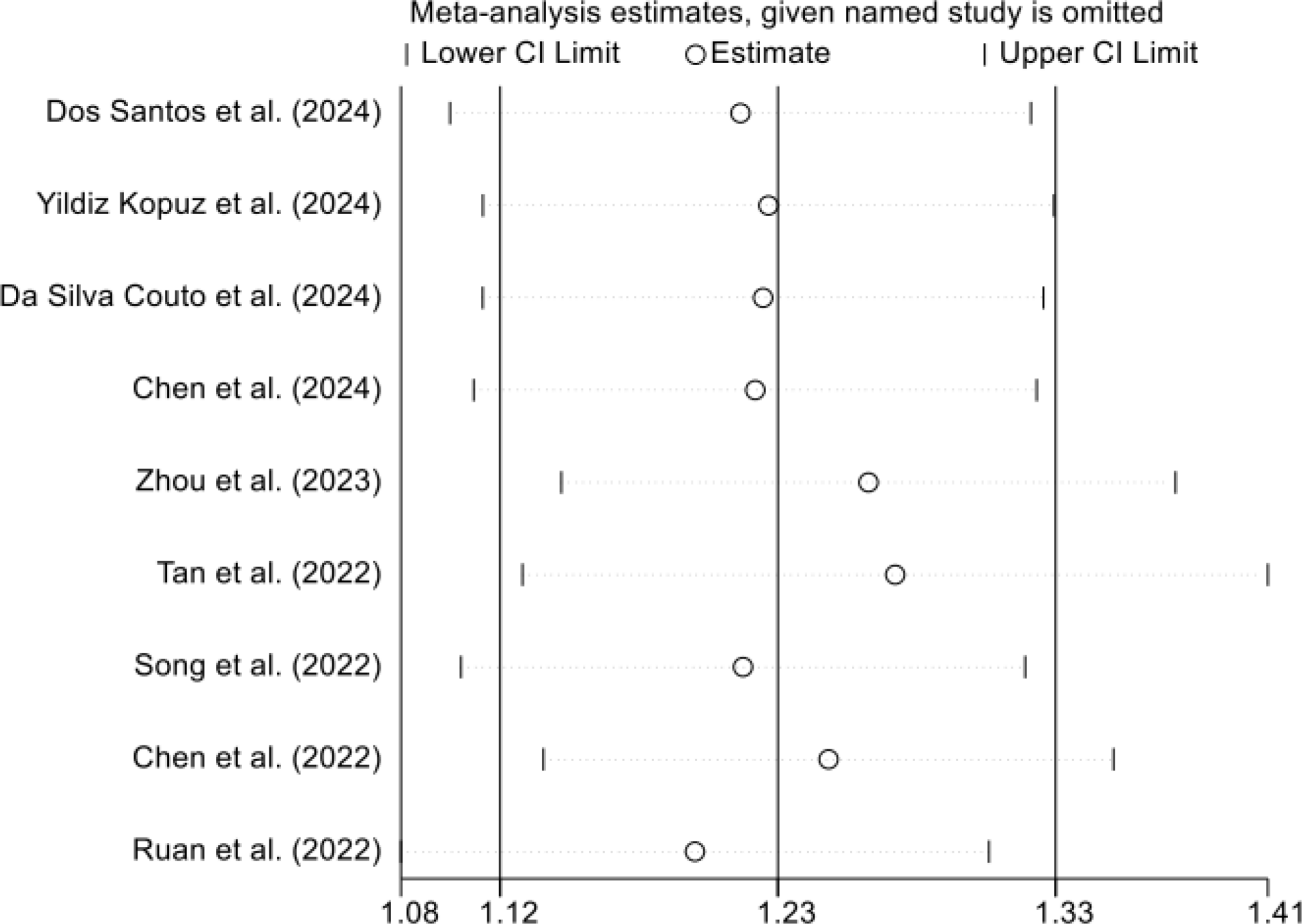
Leave-one-out sensitivity analysis evaluating the influence of each individual study on the pooled hazard ratio for overall survival.

Given the observed heterogeneity, sensitivity analyses were conducted using a leave-one-out approach. Each study was sequentially removed, and the pooled hazard ratio was recalculated using the remaining studies. Across all iterations, the direction and magnitude of the association were materially unchanged, and no single study disproportionately influenced the overall effect size (**Figure 3**). These results support the robustness of the primary analysis and suggest that the association between GLIM-defined malnutrition and overall survival was consistent across the included cohorts despite differences in study design, patient characteristics, treatment modalities, and follow-up duration.

### 3.5 Postoperative infectious complications

**Figure 4:**
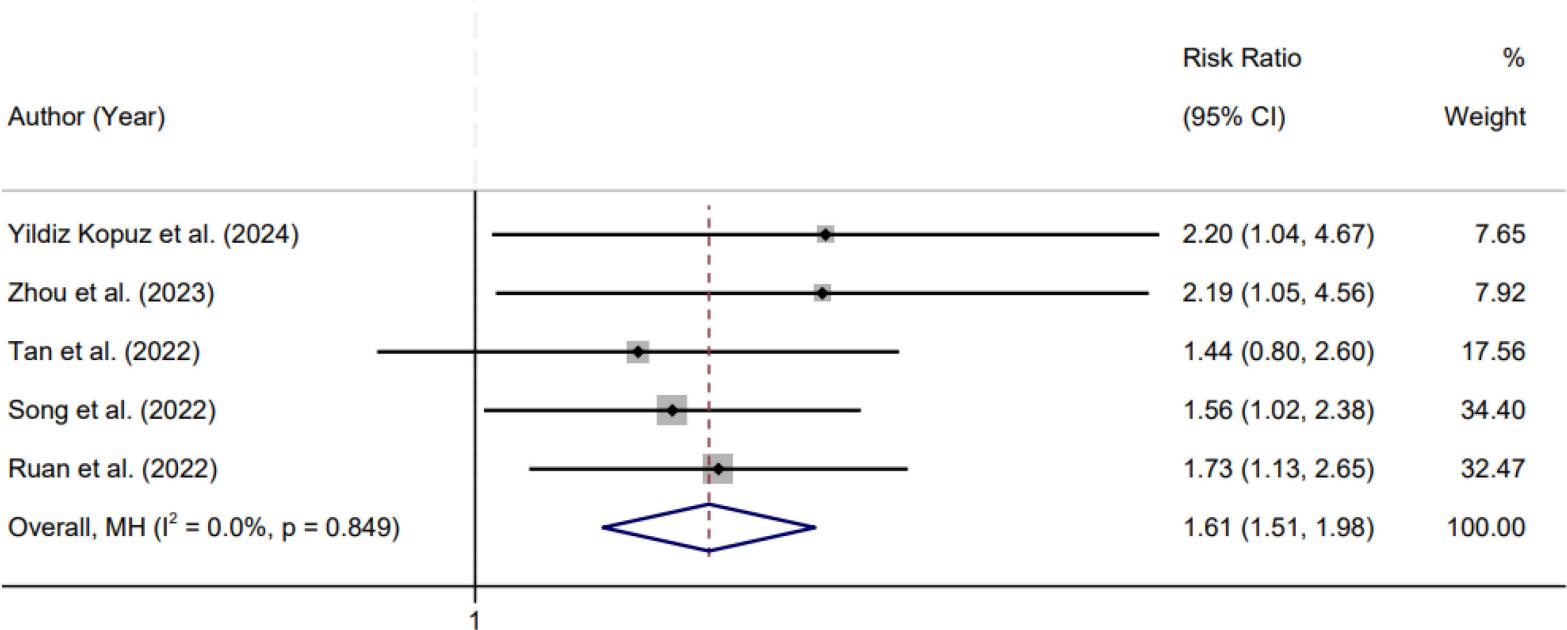
Forest plot of the association between GLIM-defined malnutrition and postoperative infectious complications in patients with colorectal cancer.

Five studies reported postoperative infectious complications and were included in the pooled analysis. Although statistical heterogeneity was not detected (I² = 0.0%, Cochran’s Q test p = 0.849), a random-effects model was applied in accordance with our prespecified analytic approach for observational studies. The meta-analysis demonstrated a significantly higher risk of postoperative infection among patients classified as malnourished according to the GLIM diagnostic criteria compared with those without GLIM-defined malnutrition. The pooled risk ratio was 1.61 (95% confidence interval 1.51–1.98) (**Figure 4**).

### 3.6 GLIM-defined malnutrition and postoperative outcomes

Malnutrition was associated with a higher risk of major complications (RR = 1.36, 95% CI 1.18–1.60) and a higher risk of overall postoperative complications (RR = 1.44, 95% CI 1.25–1.56). Short-term mortality within 30 days was also increased in the malnourished group (RR = 2.03, 95% CI 1.35–3.31), and 30-day readmission occurred more frequently among malnourished patients (RR = 1.34, 95% CI 1.01–1.64). For healthcare utilization, malnutrition was associated with a longer length of hospital stay, with a pooled mean difference of 2.30 days (95% CI 1.40–3.10). Across these outcomes, heterogeneity was low for complication and short-term event endpoints, whereas length of stay showed moderate heterogeneity (**Table 3**).

**Table 3:** Pooled postoperative outcomes comparing GLIM-defined malnutrition versus non-malnutrition.

Outcome	No. of studies (k)	Effect measure	Model	I² (%)	Q-test p value	Pooled effect (95% CI)
Major complications	5	RR	Random-effects	18.5	0.296	1.36 (1.18–1.60)
Any postoperative complications	6	RR	Random-effects	24.0	0.241	1.44 (1.25–1.56)
Length of hospital stay	4	MD, days	Random-effects	52.0	0.094	2.30 (1.40–3.10)
30-day mortality	3	RR	Random-effects	0.0	0.812	2.03 (1.35–3.31)
30-day readmission	4	RR	Random-effects	9.0	0.383	1.34 (1.01–1.64)

CI, confidence interval; GLIM, Global Leadership Initiative on Malnutrition; I², inconsistency index; k, number of studies; MD, mean difference; RR, risk ratio.

### 3.7 Subgroup analyses for overall survival and postoperative infectious complications

Subgroup analyses demonstrated that the associations of GLIM-defined malnutrition with both overall survival and postoperative infectious complications were consistent across prespecified strata (**Table 4**). For overall survival, comparable pooled effects were observed in prospective studies (HR 1.20, 95% CI 1.08–1.33; I² = 35.0%) and retrospective studies (HR 1.25, 95% CI 1.12–1.39; I² = 60.0%), with no evidence of a subgroup difference (p = 0.581). Similar results were obtained when stratified by screening approach: studies using NRS-2002 ≥3 showed an HR of 1.24 (95% CI 1.12–1.37; I² = 58.0%), while studies using other tools or not reporting screening yielded an HR of 1.20 (95% CI 1.06–1.36; I² = 41.0%) (p = 0.739). Estimates were also consistent by tumor location (rectum-only HR 1.28 vs mixed CRC HR 1.22; p = 0.662) and study quality (NOS 9 HR 1.24 vs NOS 8 HR 1.21; p = 0.827). For postoperative infectious complications, increased risk was observed across all strata with no detectable heterogeneity within subgroups (I² = 0.0%). Pooled RRs were similar between prospective and retrospective studies (RR 1.58 vs 1.79; p = 0.570), between screening categories (NRS-2002 ≥3 RR 1.65; p = 0.963), between tumor location strata (rectum-only RR 1.76 vs mixed CRC RR 1.69; p = 0.865), and between NOS categories (RR 1.61 vs 1.77; p = 0.677).

**Table 4:** Subgroup analyses for overall survival and postoperative infectious complications.

Outcome	Subgroup factor	Level	Studies (k)	Model	Pooled effect (95% CI)	I² (%)	p for subgroup difference
Overall survival	Study design	Prospective	3	Random-effects	HR 1.20 (1.08–1.33)	35.0	0.581
		Retrospective	6	Random-effects	HR 1.25 (1.12–1.39)	60.0	
	Screening tool	NRS-2002 ≥3	6	Random-effects	HR 1.24 (1.12–1.37)	58.0	0.739
		Other or not reported	3	Random-effects	HR 1.20 (1.06–1.36)	41.0	
	Tumor location	Rectum-only	2	Random-effects	HR 1.28 (1.10–1.48)	0.0	0.662
		Mixed CRC	6	Random-effects	HR 1.22 (1.10–1.34)	59.0	
	Study quality	NOS = 9	4	Random-effects	HR 1.24 (1.12–1.38)	44.0	0.827
		NOS = 8	5	Random-effects	HR 1.21 (1.09–1.35)	61.0	
Postoperative infectious complications	Study design	Prospective	2	Random-effects	RR 1.58 (1.23–2.20)	0.0	0.570
		Retrospective	3	Random-effects	RR 1.79 (1.25–2.36)	0.0	
	Screening tool	NRS-2002 ≥3	4	Random-effects	RR 1.65 (1.38–2.16)	0.0	0.963
		Other or not reported	1	Random-effects	RR 1.67 (0.98–2.45)	—	
	Tumor location	Rectum-only	2	Random-effects	RR 1.76 (1.33–2.56)	0.0	0.865
		Mixed CRC	3	Random-effects	RR 1.69 (1.15–2.25)	0.0	
	Study quality	NOS = 9	2	Random-effects	RR 1.61 (1.27–2.21)	0.0	0.677
		NOS = 8	3	Random-effects	RR 1.77 (1.19–2.39)	0.0	

CI, confidence interval; CRC, colorectal cancer; GLIM, Global Leadership Initiative on Malnutrition; HR, hazard ratio; I², inconsistency index; k, number of studies; NOS, Newcastle–Ottawa Scale; NRS-2002, Nutritional Risk Screening 2002; RR, risk ratio.

### 3.8 Publication bias assessment

**Figure 5:**
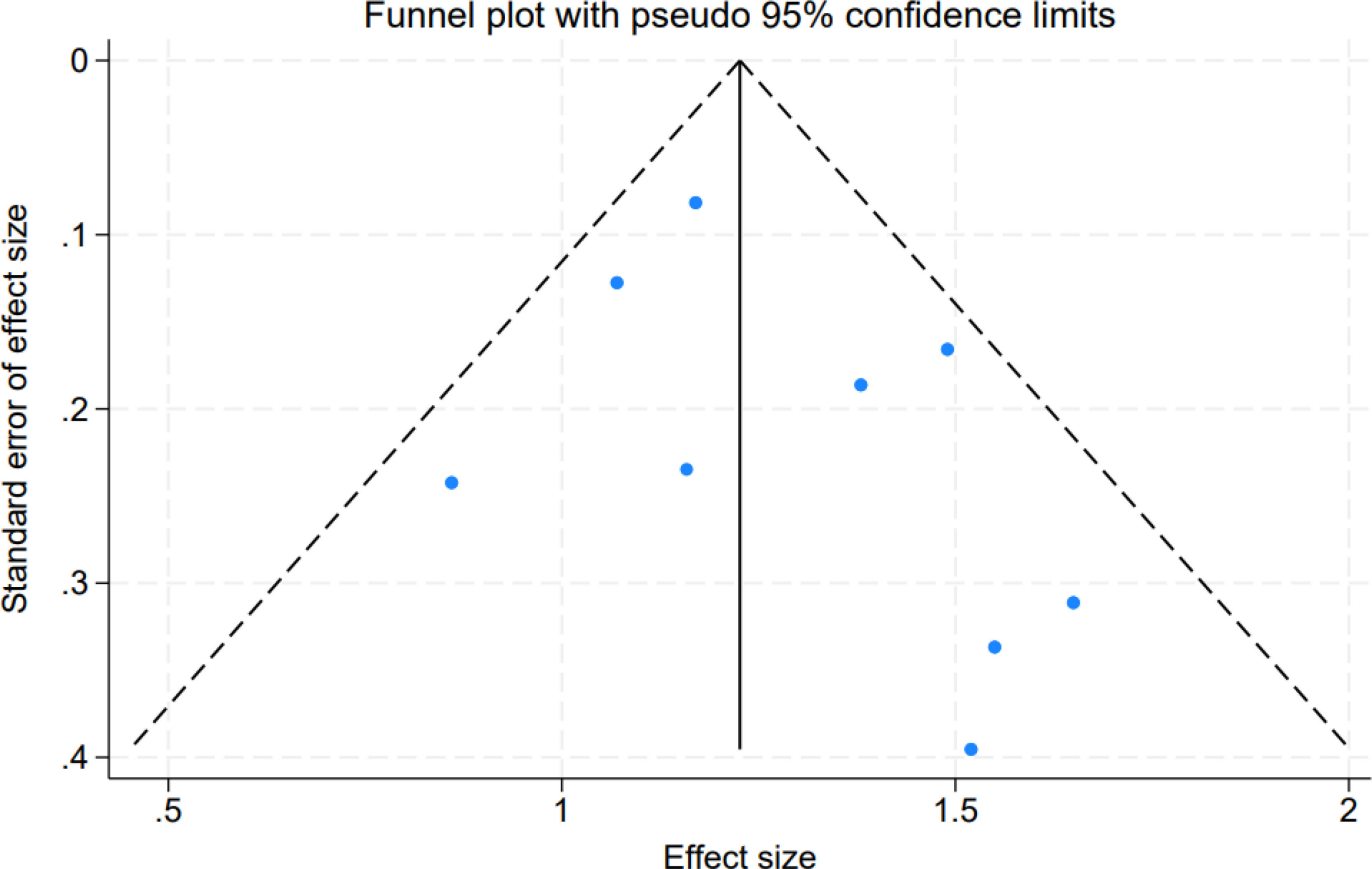
Funnel plot assessing small-study effects and potential publication bias for the primary meta-analytic outcomes.

Potential publication bias was evaluated using visual inspection of funnel plots and Egger’s linear regression test. The funnel plots did not show marked asymmetry and appeared generally symmetric, suggesting no obvious small-study effects (**Figure 5**). Consistently, Egger’s regression tests were not statistically significant for any pooled outcome, indicating no evidence of publication bias (all P values > 0.05).

## 4 Discussion

This systematic review and meta-analysis evaluated the association between malnutrition and prognosis in colorectal cancer using a standardized diagnostic framework. The principal finding was that GLIM-defined malnutrition was associated with inferior overall survival, with a pooled hazard ratio of 1.23, indicating an increased hazard of death during follow-up among malnourished patients. This association remained stable in leave-one-out analyses and persisted across prespecified subgroups, supporting the internal robustness of the survival signal. In addition to long-term prognosis, GLIM-defined malnutrition was associated with short-term postoperative vulnerability. Malnourished patients had a higher risk of postoperative infectious complications (RR 1.61, 95% CI 1.51–1.98), as well as higher risks of major complications (RR 1.36, 95% CI 1.18–1.60) and overall postoperative complications (RR 1.44, 95% CI 1.25–1.56), higher 30-day mortality (RR 2.03, 95% CI 1.35–3.31), increased 30-day readmission (RR 1.34, 95% CI 1.01–1.64), and a longer length of stay (mean difference 2.30 days, 95% CI 1.40–3.10). Collectively, these findings are coherent with the clinical construct that malnutrition reflects reduced physiologic reserve and impaired host defense in patients undergoing complex oncologic treatment (32). Mechanistically, GLIM-defined malnutrition integrates phenotypic criteria (unintentional weight loss, low body mass index, and reduced muscle mass) and etiologic criteria (reduced intake or absorption, and inflammation or disease burden). These domains map onto pathways that plausibly influence both survival and perioperative outcomes (33). Reduced skeletal muscle mass and functional impairment can be associated with lower cardiopulmonary reserve, delayed mobilization, and reduced tolerance to surgical stress, while reduced intake and systemic inflammation may contribute to impaired tissue repair and dysregulated immune responses (34). In the perioperative setting, these factors may increase susceptibility to infectious complications, which can prolong hospitalization, increase readmissions, and interrupt adjuvant treatment trajectories (35). Over longer horizons, malnutrition may also correlate with reduced tolerance or completion of chemotherapy and radiotherapy, resulting in less effective dose intensity and downstream survival differences (36, 37). While this meta-analysis was not designed to establish causality, the observed pattern across survival, complication, and utilization endpoints is consistent with a clinically meaningful nutritional phenotype relevant to both oncologic and surgical pathways (38).

The direction and clinical interpretation of the present results align with emerging colorectal cancer–specific evidence and broader oncologic surgery data published in recent years. A colorectal cancer–focused meta-analysis published in 2025 reported that GLIM-defined malnutrition was associated with worse overall survival and increased postoperative complications, with infection-related complications identified as a particularly relevant outcome domain (39). This convergence across independent syntheses supports the reproducibility of the association when GLIM is applied within colorectal cancer cohorts. Furthermore, an observational study in elderly patients with colorectal cancer found that GLIM-defined malnutrition was associated with postoperative complications and decreased overall survival, which is directionally consistent with the pooled estimates observed in the present review (13, 40). The prognostic relevance of GLIM has also been explored in specific clinical phenotypes prone to under-recognition, including overweight patients in whom low muscle mass and reduced function may coexist with preserved or elevated body mass. A 2024 study evaluating GLIM combined with handgrip strength in overweight colorectal cancer patients emphasized that malnutrition may be overlooked in this subgroup and linked nutritional classification to prognosis, supporting the need for structured assessment beyond body mass index alone (41, 42). Beyond single cohorts, recent work has examined implementation strategies and screening alignment. For example, a 2024 study published in Clinical Nutrition reported that NRS-2002 performed well as a screening tool to support GLIM diagnosis in colorectal cancer and that GLIM-based classification was associated with adverse prognosis, providing context for why NRS-2002 was the predominant screening approach across the included cohorts (43). At the level of surgical outcomes across malignancies, a large international study published in 2023 found that malnutrition was common and associated with increased 30-day mortality and surgical-site infection after elective cancer surgery, which is consistent with the significantly increased risks of postoperative infectious complications and short-term mortality observed in our analysis (4). Taken together, recent colorectal cancer–specific and broader oncologic evidence supports the clinical plausibility of GLIM-defined malnutrition as a prognostic marker and perioperative risk stratifier.

Moderate heterogeneity was observed for overall survival (I² = 56.4%), whereas no detectable statistical heterogeneity was identified for postoperative infectious complications (I² = 0.0%). Heterogeneity was also low for major complications, overall postoperative complications, short-term mortality, and 30-day readmission, while length of stay showed moderate heterogeneity. Several factors may explain this pattern. First, overall survival integrates heterogeneous treatment trajectories and competing risks across study settings, including differences in stage distribution, oncologic treatment modalities, and access to perioperative optimization. These sources of variability can influence both baseline prognosis and the degree to which nutritional status translates into survival differences (44). Second, the operationalization and timing of nutritional assessment may differ across cohorts. Some studies may have applied GLIM classification at admission, preoperatively, or during treatment, and the clinical meaning of malnutrition may vary across these time points (45). Third, although GLIM provides a standardized framework, the specific measurement methods used to define reduced muscle mass and the thresholds applied for phenotypic criteria may vary, introducing non-differential misclassification that could attenuate or diversify effect sizes across cohorts (46). Fourth, confounding control was likely heterogeneous. Although most studies achieved strong comparability scores on the NOS, covariate sets and modeling strategies were not identical; residual confounding by disease severity, inflammation, comorbidity burden, and treatment intensity may therefore remain (47). The subgroup analyses did not identify statistically significant effect modification by study design, screening tool category, tumor location, or NOS score for either overall survival or postoperative infectious complications. This suggests that the direction of association was stable across prespecified strata, whereas the residual heterogeneity observed for survival may reflect unmeasured or incompletely harmonized factors, including differences in staging, perioperative pathways, and the timing and intensity of nutritional support (48). For postoperative infectious complications, the absence of detectable heterogeneity suggests that the adverse association of GLIM-defined malnutrition was relatively consistent across studies, although a random-effects model was still applied in accordance with the prespecified analytic strategy for observational studies. For length of stay, moderate heterogeneity is expected because discharge criteria and health system factors vary substantially across centers and regions, and these system-level differences can influence utilization outcomes even when clinical complication rates are comparable (49).

The present findings support routine nutritional risk screening followed by GLIM-based diagnostic assessment in colorectal cancer care pathways, particularly in the context of surgical decision-making and perioperative planning. Across the included studies, GLIM diagnosis was generally established by combining at least one phenotypic criterion (e.g., unintentional weight loss, low body mass index, or reduced muscle mass assessed by computed tomography–derived skeletal muscle index or handgrip strength) with one etiologic criterion, most commonly inflammation or disease burden related to colorectal cancer (29, 50). Identification of GLIM-defined malnutrition may therefore inform risk communication, perioperative optimization, and multidisciplinary care planning. Given the significantly increased risks of postoperative infectious complications, major complications, overall postoperative complications, and short-term mortality observed in this review, targeted preoperative nutritional support and closer perioperative monitoring may be appropriate for patients meeting GLIM criteria, integrated with existing enhanced recovery and infection-prevention pathways (51). The associations with longer length of stay and higher 30-day readmission further suggest potential value for discharge planning and for stratifying the intensity of post-discharge follow-up according to nutritional status (52).

This study has several strengths. The review was conducted in accordance with PRISMA guidance, synthesized evidence from observational cohort studies using a standardized malnutrition framework, and focused on clinically actionable outcomes spanning survival, postoperative complications, and healthcare utilization. The overall methodological quality of the included cohorts was high based on NOS scoring, and the primary survival finding was supported by sensitivity analyses showing that no single study disproportionately influenced the pooled association. Subgroup analyses further demonstrated consistency of the observed associations across key study-level characteristics, and visual inspection of funnel plots together with Egger’s testing did not suggest substantial publication bias. Several limitations should also be acknowledged. First, the evidence base consisted exclusively of observational cohorts, which limits causal inference and leaves open the possibility of residual confounding by disease burden, inflammation, frailty, comorbidities, and treatment selection. Second, although GLIM provides a common diagnostic framework, between-study differences in the operationalization of phenotypic criteria, particularly the assessment of reduced muscle mass, may have introduced misclassification and contributed to heterogeneity, especially for overall survival. Third, several outcomes were reported by only a small number of studies, restricting precision and limiting the reliability of subgroup difference tests. Fourth, definitions and ascertainment windows for postoperative infections and complications may have varied across cohorts, potentially affecting absolute event rates and contributing to between-study differences even when the overall direction of association remained consistent. Finally, the review could not consistently evaluate dose–response relationships across GLIM severity categories or quantify the effects of nutritional interventions, because these data were not uniformly reported. Future research should focus on validating the applicability and consistency of the GLIM criteria across different regions and healthcare settings, with emphasis on further standardizing its implementation. Large-scale, multicenter, long-term prospective studies are needed to better define the long-term impact of malnutrition on colorectal cancer outcomes. In addition, future studies should determine whether targeted nutritional interventions guided by GLIM-defined malnutrition can improve clinical outcomes, particularly in perioperative and systemic treatment settings.

## 5 Conclusions

GLIM-defined malnutrition was associated with adverse prognosis in colorectal cancer. Across nine cohorts, malnourished patients had poorer overall survival, with findings remaining robust in sensitivity analyses. Malnutrition was also associated with higher postoperative infectious complications, increased major complications, increased overall postoperative complications, higher 30-day mortality, higher 30-day readmission, and longer hospitalization. Subgroup analyses showed generally consistent associations across study-level characteristics.

## Data Availability

The raw data supporting the conclusions of this article will be made available by the authors, without undue reservation.
