# Supplementary material for: Association between malnutrition and prognosis in colorectal cancer: a systematic review and meta-analysis
Source: Front Oncol. 2026 May 8;16:1789366. doi: 10.3389/fonc.2026.1789366 (PMC13193982; doi:10.3389/fonc.2026.1789366)
Supplement: Supplementary file 1 [file Table1.docx]

Supplementary Table 1. Detailed search strategies.

| Database | Search strategy (controlled vocabulary + free-text; adapted per platform) |
| --- | --- |
| PubMed | #1 Colorectal cancer terms: (“Colorectal Neoplasms”[MeSH] OR “Colon Neoplasms”[MeSH] OR “Rectal Neoplasms”[MeSH] OR colorectal cancer*[Title/Abstract] OR colon cancer*[Title/Abstract] OR rectal cancer*[Title/Abstract] OR colorectal neoplasm*[Title/Abstract] OR colon neoplasm*[Title/Abstract] OR rectal neoplasm*[Title/Abstract] OR CRC[Title/Abstract]) #2 GLIM/malnutrition terms: (“Malnutrition”[MeSH] OR malnutrition[Title/Abstract] OR undernutrition[Title/Abstract] OR “nutritional status”[Title/Abstract] OR “Global Leadership Initiative on Malnutrition”[Title/Abstract] OR GLIM[Title/Abstract]) #3 Prognosis terms: (“Prognosis”[MeSH] OR “Survival”[MeSH] OR “Treatment Outcome”[MeSH] OR prognos*[Title/Abstract] OR surviv*[Title/Abstract] OR mortality[Title/Abstract] OR death[Title/Abstract] OR “overall survival”[Title/Abstract] OR OS[Title/Abstract] OR “disease-free survival”[Title/Abstract] OR DFS[Title/Abstract] OR recurrence[Title/Abstract] OR “progression-free survival”[Title/Abstract] OR PFS[Title/Abstract] OR outcome*[Title/Abstract]) #4 Study design filter (cohort): (cohort[Title/Abstract] OR “cohort studies”[MeSH] OR prospective[Title/Abstract] OR retrospective[Title/Abstract] OR longitudinal[Title/Abstract] OR “follow-up”[Title/Abstract]) Final: #1 AND #2 AND #3 AND #4 |
| Embase | #1 Colorectal cancer terms: (‘colorectal cancer’/exp OR ‘colon cancer’/exp OR ‘rectum cancer’/exp OR colorectal:ti,ab OR colon:ti,ab OR rectal:ti,ab AND (cancer*:ti,ab OR neoplasm*:ti,ab OR tumor*:ti,ab OR carcinoma*:ti,ab) OR CRC:ti,ab) #2 GLIM/malnutrition terms: (‘malnutrition’/exp OR malnutrition:ti,ab OR undernutrition:ti,ab OR ‘nutritional status’:ti,ab OR ‘global leadership initiative on malnutrition’:ti,ab OR GLIM:ti,ab) #3 Prognosis terms: (‘prognosis’/exp OR ‘survival’/exp OR ‘treatment outcome’/exp OR prognos*:ti,ab OR surviv*:ti,ab OR mortality:ti,ab OR recurrence:ti,ab OR outcome*:ti,ab OR ‘overall survival’:ti,ab OR ‘disease free survival’:ti,ab OR ‘progression free survival’:ti,ab) #4 Cohort terms: (‘cohort analysis’/exp OR ‘cohort study’/exp OR cohort*:ti,ab OR prospective:ti,ab OR retrospective:ti,ab OR longitudinal:ti,ab OR ‘follow up’:ti,ab) Final: #1 AND #2 AND #3 AND #4 |
| Web of Science Core Collection | TS=((colorectal OR colon OR rectal) NEAR/2 (cancer* OR neoplasm* OR tumor* OR carcinoma*) OR “colorectal neoplasm*” OR CRC) AND TS=(malnutrition OR undernutrition OR “nutritional status” OR “Global Leadership Initiative on Malnutrition” OR GLIM) AND TS=(prognos* OR surviv* OR mortality OR recurrence OR outcome* OR “overall survival” OR “disease-free survival” OR “progression-free survival”) AND TS=(cohort OR prospective OR retrospective OR longitudinal OR “follow-up”) |
| The Cochrane Library | (colorectal OR colon OR rectal):ti,ab,kw AND (cancer* OR neoplasm* OR tumor* OR carcinoma*):ti,ab,kw AND (malnutrition OR undernutrition OR “nutritional status” OR “Global Leadership Initiative on Malnutrition” OR GLIM):ti,ab,kw AND (prognos* OR surviv* OR mortality OR recurrence OR outcome* OR “overall survival” OR “disease-free survival” OR “progression-free survival”):ti,ab,kw |

Notes for implementation:

The search strings above are intended to be database-specific templates; field tags and proximity operators should be adjusted according to each platform’s syntax (e.g., PubMed [Title/Abstract] vs. Embase ti,ab vs. Web of Science TS).

If the initial yield is small, consider removing the cohort filter (#4) and screening study designs during selection to maximize sensitivity, while retaining the core concept blocks (colorectal cancer AND GLIM/malnutrition AND prognosis).
